# Custodians of Controversy: Navigating Stewardship Challenges With Non‐Consensual Anatomized Human Skeletonized Individuals in South Africa

**DOI:** 10.1002/ajpa.70300

**Published:** 2026-07-02

**Authors:** A. Alblas, V. E. Gibbon

**Affiliations:** ^1^ Department of Biomedical Sciences, Faculty of Medicine and Health Sciences Stellenbosch University Cape Town South Africa; ^2^ Department of Human Biology, Division of Clinical Anatomy & Biological Anthropology University of Cape Town Cape Town South Africa

**Keywords:** anatomical donation programs, anatomical education, body donation, cadaveric, curator perspective, ethics, skeletal collections, unclaimed

## Abstract

South Africa, like many countries, holds collections of human skeletonized individuals historically acquired through anatomical dissection programs. This paper examines non‐consensual anatomized and unclaimed (NCAU) state‐directed skeletonized individuals, whose presence in collections pose significant legal, ethical, and professional challenges. Although the National Health Act (2003) outlines procedures for body donation, the inclusion of unclaimed and destitute persons obtained without consent raises ethical concerns. As curators and custodians, a perspective is provided, assessing the legal and ethical implications of retaining NCAU skeletonized individuals and highlighting inconsistencies between South African regulations and international standards that prioritize informed consent and dignity‐in‐death. The complexities surrounding the retention, use, or disposition of these decedent bodies, and parts thereof, are discussed, offering recommendations for institutional decision‐making to navigate these complexities. A structured and multidisciplinary approach is provided we advocate for transparent and ethically responsible stewardship of NCAU collections. Furthermore, the pressure placed on curators is noted, who face potential personal and professional repercussions, and emphasizes the need for institutional support, training, and resources to guide ethical management and facilitate informed decision‐making of these sensitive collections.

## Research Highlights

- Non‐consensual anatomized and unclaimed state‐directed repositories of human skeletonized individuals in South Africa raise complex ethical concerns despite legal retention.
- A moratorium offers time for inclusive dialogue and ethical decision‐making.
- Guidelines for committee formation and institutional responsibility support ethical curation and curator protection.

## 1 Introduction

At the outset of this paper, a rationale and set of definitions are provided for curatorial terminology used in the manuscript, which may differ in other countries or institutions. A group of skeletonized human individuals is known broadly as a collection, whether housed in a formalized repository or not; it can refer to any group of skeletonized individuals gathered for study or display, regardless of how they are stored or managed. The term *collection* often emphasizes the individual pieces or samples and has an association with ownership, and thus, linked to heritage laws and collection management, rather than the system of care or curation; it also speaks to a historical era of the act of collecting in museological spaces (Rassool and Gibbon 2024). Whereas a *repository* in this context typically refers to an organized, often formalized storage space or facility where human skeletonized individuals (or other types of biological samples) are kept for research, education, or preservation. It is often based at an institution (museum, university, or research facility) that serves as its custodian. Therefore, the term repository is used as a shift in thinking toward responsible stewardship and away from the practice of collecting. Stewardship emphasizes accountability, sustainability, and ethical responsibility in the use and protection of resources, whether they are natural, financial, or cultural. A *custodian* is a person responsible for caring, protecting, or maintaining something on behalf of others. This may involve tangible assets (property, documents, or funds) or intangible assets (culture or traditions). Custodians often have a legal or ethical obligation to ensure the safety and preservation of what is entrusted to them. Whereas the person responsible for the management, organization, and care of a collection, such as in a museum, gallery, or archive, or in this case, skeletonized individuals at a university, is often referred to as a *curator*. The role involves selecting, organizing, and overseeing exhibits, as well as ensuring the preservation of the collection/repository. A repository may imply a collection that has been curated and documented under specific ethical, legal, and professional guidelines. Custodians and curators engage in stewardship‐responsible management, supervision, or care of resources, assets, or duties that are entrusted to an individual or group.

As human anatomy and biological anthropology's ethical standards change, custodians and curators, the people behind contemporary stewardship, must navigate complex and multifaceted ethical considerations and stakeholder expectations. Custodians of repositories in museums and universities around the world are navigating difficult collecting practices of the past, which often reflect the collector (person who collected, accessioned, or curated), sociocultural practices of the period, region, and discipline. The ethical concerns surrounding these collections are associated with contentious and problematic histories such as colonialism, racial science, unethical acquisition and exploitation of marginalized communities (Richardson 2001; Mukhopadhyay and Henze 2003; Sealy 2003; Garment et al. 2007; Halperin 2007; Hildebrandt 2008, 2022; Morris 2009; Jones and Whitaker 2012; Nystrom 2017; Legassick and Rassool 2015; Ghosh 2015; Schramm 2016; Jones 2019, 2023; Rassool and Gibbon 2024; Rassool and Hayes 2021; Zuckerman et al. 2021; Black et al. 2022; Stantis et al. 2023; Agarwal 2024; Agarwal et al. 2024; Baliso et al. 2025). Although debates on unethical collections of human skeletonized individuals often center on archaeological and historical contexts, those derived from anatomical education programs raise similar concerns.

At present, there is no centralized international and national registry that systematically differentiates collections of human skeletonized individuals by provenance, such as willed donation, historical acquisition, cemetery‐based recovery, or non‐consensual anatomized and unclaimed (NCAU) state‐directed, nor one that verifies their current status, scale, or governance structures. The Forensic Anthropology Society of Europe (FASE) has made a significant effort to document and map known osteological collections globally (Petaros et al. 2021). FASE currently identifies approximately 150 documented collections of human skeletonized individuals worldwide, distributed across diverse national and institutional settings (see de la Cova 2012; FASE n.d.). A small but significant subset, estimated at approximately 30 to 50 collections, was developed primarily through structured anatomical body donation programs, including the United States, Australia, South Africa, and selected countries in Asia, Europe, and South America. The presence of individuals sourced from unclaimed deceased persons, anatomical dissection, or forensic contexts complicates assuming consent‐based provenance.

Many historically significant collections of human skeletonized individuals, including the Robert J. Terry and Hamann‐Todd collections in the United States, are contemporaneously affected by ethically problematic sourcing rooted in historical standards and consent frameworks (Hunt and Albanese 2005). Comparable patterns are evident in South African medical school collections, such as the Pretoria Bone Collection, Raymond A. Dart Collection of Human Skeletons, Stellenbosch Bone Repository, and the UCT Human Skeletal Repository (Kramer et al. 2019; Baliso et al. 2025). While these repositories remain vital for demographic, comparative, and methodological research, their foundations outside contemporary informed consent frameworks continue to raise ethical concerns regarding their retention, governance, and ongoing use.

Curators confront stewardship dilemmas that echo global debates, balancing the scientific value of these collections with the ethical legacies of their acquisition. As South African curators and custodians, we face challenges and complexities in the retention, use, disposition, and stewardship of skeletonized individuals of NCAU state‐directed origin, which are shaped by the intersection of historical inequities and contemporary ethical scrutiny. Beyond legal compliance, these collections foreground broader concerns relating to consent, dignity, historical marginalization, and the ethical boundaries of scientific stewardship (Richardson 2001; Gangata et al. 2010; Nystrom 2017; Muller et al. 2017; Alves‐Cardoso 2019; de la Cova 2020a, 2020b, 2022; Campanacho et al. 2021; Zuckerman et al. 2021; Hildebrandt 2022; Plens et al. 2022; Agarwal 2024; Manjatika et al. 2024). A series of considerations and guidelines for navigating these curatorial complexities are presented.

### 1.1 Stewardship Challenges for Anatomically Derived Repositories of Human Skeletonized Individuals in South Africa

Anatomical dissection programs have long depended on human body donations for medical and health sciences training, and in the early twentieth century this reliance often resulted in controversial and unethical sourcing practices (Jones 1994, 2019, 2023; Richardson 2001; Tward and Patterson 2002; Davidson 2007; Garment et al. 2007; Halperin 2007; Hildebrandt 2009; Champney 2011; Jones and Whitaker 2012; Ghosh 2015; Schramm 2016; Winkelmann 2016; Habicht et al. 2018; Comer 2022; Agarwal et al. 2024). The use of human bodies in medical schools, hospitals, or research facilities can occur through personal donation or state mechanisms. The latter category, NCAU skeletonized persons, raises significant ethical concerns as they are obtained without explicit consent from the individual before death, or their family, or legal representatives; the body and any tissue thereof could be retained for long‐term curation (sometimes indefinitely), which was used for health sciences education and research (Gangata et al. 2010). Retention of the skeleton or bones was a common and regular practice in South Africa and has led to large anatomically derived repositories of human skeletonized individuals, with NCAU skeletonized individuals (L'Abbé et al. 2005, 2021; Dayal et al. 2009; Alblas et al. 2018; Maass and Friedling 2019; Gibbon and Morris 2021; Maass 2023; Baliso et al. 2025). In South Africa, more than half of ~6200 human skeletons in repositories originate from anatomical donation programs (Baliso et al. 2025). Collections of human skeletonized individuals produced through such practices pose complex challenges for contemporary custodians, particularly when these NCAU skeletonized individuals are in long‐term institutional care; they challenge principles of human dignity, autonomy, and informed consent. A central point of contention is curatorship itself. In South Africa, the stewardship of repositories of human skeletonized individuals is shaped by the intersecting legacies of colonialism, apartheid, and structural violence.

The provenance of South African anatomical repositories is entangled with the racialized hierarchies and systemic inequalities of apartheid (1948–1994) (May 1998; Ntatamala et al. 2023). During this period, individuals defined in the South African census as “black‐African” or “colored” were disproportionately represented among the destitute, unclaimed, and institutionally confined. These individuals were more likely to be funneled into state anatomical programs under the guise of legal sanction. International scholarship has identified that the acquisition of human bodies from marginalized communities often reflects systemic vulnerabilities, poverty, ill‐health, oppression, dispossession and discrimination, that extend beyond the individual to entire social groups (Nystrom 2017; Forde et al. 2019; Halperin 2007; M'charek et al. 2020; Zuckerman et al. 2021; Molina‐Pérez et al. 2022; de la Cova et al. 2024). In South Africa, these vulnerabilities were amplified by apartheid's legislative frameworks, which institutionalized inequality across every aspect of life (Jansen and Walters 2020; Gumede 2021; Pellicer and Ranchhod 2023; Ntatamala et al. 2023). While state‐sanctioned, the accrual of deceased persons from these sources raises ethical concerns in the post‐apartheid era, where the echoes of colonial exploitation and systemic injustice continue to shape lived social realities of South Africans. Thus, these collections, like others found elsewhere, embody marginalization and structural inequality (Nystrom 2017; Alves‐Cardoso 2019; de la Cova 2020a, 2020b, 2022; Campanacho et al. 2021; Zuckerman et al. 2021; IFAA 2022; Watkins 2022; de la Cova et al. 2023, 2024; Kim and Friedlander 2023; Stantis et al. 2023; Agarwal 2024; Agarwal et al. 2024; Smithsonian Human Remains Task Force 2024; Auerbach and Jackson 2022).

Custodians of NCAU collections/repositories of human skeletonized individuals face ethical, legal, and professional challenges rooted in the conditions under which they were acquired. Effective stewardship demands reckoning with these legacies, recognizing the vulnerability of the individuals represented, and ensuring they are treated with sensitivity, care, and respect; failing to do so risks perpetuating the harm inherent in their acquisition. This context situates South African repositories of human skeletonized individuals within broader global debates on structural violence, stewardship, and curatorship. As South African curators and custodians, a local perspective is provided on the legal, ethical, and professional challenges involved in stewarding NCAU repositories of human skeletonized individuals acquired through anatomical body donation. South African practices are situated alongside international institutional guidance and ethical scholarship on structural violence and stewardship.

## 2 Legality for NCAU Skeletonized Individuals Derived Through Anatomical Education Programs in South Africa

Awareness of South Africa's legislation on anatomical body donation is essential for understanding repositories of human skeletonized individuals derived from these programs. Anatomical body donation is regulated by health authorities and overseen by the Health Officer: Inspectorate of Anatomy in provinces where the position is active. Initially, it was governed by the South African Human Tissue Act of 1911 and the Anatomy Act (No. 20 of 1959). These laws were updated in 1983 with the South African Human Tissue Act (No. 65 of 1983) and further refined by the National Health Act of South Africa of 2003 and its 2013 amendment (NHA‐SA). We refer to personal donation as bodies donated under the NHA‐SA in section 62.1.a or 62.2 by direct self, family, or next‐of‐kin (legal representative) body donation after their death. These persons' bodies are donated under informed consent willingly and voluntarily for health sciences education and research. In contrast, under the NHA‐SA section 62.3, government (state) institutions may direct the bodies of unclaimed and destitute persons (sometimes referred to as paupers) in hospitals, or individuals convicted of crimes who have died in prisons, to universities for anatomical educational programs without the individual's consent (Jones 1994; Richardson 2001; Hildebrandt 2008, 2022; Jones and Whitaker 2012).

Under 17 of the Regulations Regarding the Rendering of Forensic Pathology Service (2018), promulgated in terms of the National Health Act 61 of (SA‐NHA‐SA 2003), unidentified or unclaimed human bodies/remains, defined as those not claimed by next‐of‐kin or responsible parties within a prescribed period (typically 30 days), may, at the discretion of the Director‐General of the Department of Health, be transferred to an authorized institution. In alignment with the NHA‐SA, this creates a legal pathway whereby these bodies are available for health sciences education and research (see sections 63 and 64(1)). These individuals, often referred to as non‐consensual, anatomized, and unclaimed (NCAU) state‐directed, lack informed consent and voluntariness from either the decedent or next‐of‐kin.

In practice, the transition to “unclaimed” occurs within a fragmented system where no centralized statutory mechanism exists to mandate or coordinate the active tracing of next‐of‐kin. Instead, responsibilities are distributed across health services, law enforcement, civil registration, and municipal authorities; this topic in detail is being explored in a separate manuscript by Alblas. While the NHA‐SA and the Regulations Relating to the Management of Human Remains (R.363 of 2013) require the respectful certification, recording, and handling of the deceased, they do not impose an explicit obligation for systematic kin‐tracing. Active identification efforts, that may facilitate locating relatives, are primarily undertaken by the South African Police Service (SAPS). In cases of unnatural or suspicious deaths, this occurs under the Criminal Procedure Act 51 of (Republic of South Africa 1977) and the Inquest Act 58 of (Republic of South Africa 1959) Union Gazette Extraordinary No. 6254 with support from forensic identification methods such as fingerprinting and DNA profiling under the Criminal Law (Forensic Procedures) Amendment Act 37 of (Republic of South Africa 2014).

For natural deaths occurring in medical institutions, the Births and Deaths Registration Act 51 of (Republic of South Africa 1992) Government Gazette No. 13953 governs death registration and burial authorization but does not establish a clear duty for proactive kin‐location beyond reasonable administrative steps. Once individuals are formally classified as unclaimed, municipal policies prioritize burial or cremation rather than continued efforts to trace relatives. Consequently, in South Africa kin‐location practices remain inconsistent and context‐dependent, disproportionately affecting marginalized, structurally vulnerable, and undocumented individuals, who may transition from “unidentified” to “unclaimed” without sustained or coordinated attempts to locate next‐of‐kin. While the (SA‐NHA‐SA 2003) provides a legal structure for body donation in the country, are these NCAU bodies acquired ethically?

## 3 Ethical Considerations for NCAU Skeletonized Individuals Derived Through Anatomical Education Programs in South Africa

Ethics and legality are socially shaped, have movable boundaries and are historically contingent (Jones 2019; Gibbon and Morris 2021; Gibbon et al. 2024). Scholarship has recently challenged the long‐standing use of decedent bodies in universities, museums and private collections, as they often emerged from structural marginalization, disproportionately involving the destitute and racialized (de la Cova 2019, 2020b; Lans 2021; Zuckerman et al. 2021; Plens et al. 2022; de la Cova et al. 2023, 2024; Stantis et al. 2023; Jones 2025; Robbin Schug et al. 2025). This recognition compels institutions to address legacies of historical injustices while reconsidering how present‐day practices may perpetuate inequities.

Several international professional bodies now explicitly advise against the use of NCAU bodies or skeletonized persons in research and teaching. The International Federation of Associations of Anatomists (IFAA 2022) recommends that body donation be based on explicit, informed, and voluntary consent, and that digital images or representations of deceased individuals be treated with the same respect and dignity as the body itself (Cornwall et al. 2024). Stantis et al. (2023) have called on biological anthropology to reassess repositories of human skeletonized individuals discipline‐wide, emphasizing provenance audits and the responsibility to confront structural violence embedded in the history of collecting. These positions are reinforced by the outcomes of the *Ethical Futures for Curation, Research, and Teaching in Biological Anthropology* workshop in November 2021, convened by the Smithsonian Institution. It brought together international scholars, curators, journal editors, and funding representatives to articulate a shared ethical framework that rejects the continued research and pedagogical use of non‐consensually acquired or non‐consensual deceased persons, and advocates for moratoria, descendant community consultation, and consent‐based governance structures where provenance or authorization is unclear (de la Cova et al. 2024). At an institutional level, the Smithsonian Human Remains Task Force (2024) recommended comprehensive provenance reviews, transparent public reporting, and formal pathways for repatriation or reburial where appropriate. Similarly, the American Anthropological Association's Commission for the Ethical Treatment of Human Remains (2024) advised establishing permanent oversight bodies, formalized community consultation, and the integration of descendant perspectives into all decisions regarding retention, research, and teaching. Additionally, de la Cova (2022) calls for paleopathologists to explicitly reposition themselves ethically, centering consent, historical context, and community engagement as foundational to the study of ancient deceased persons, and moving the discipline toward practices that prevent further harm to descendants and honor the lived identities of those studied. Contemporary curators in South Africa should strive to the same thinking. Together, these statements articulate a global ethical consensus: legality alone does not justify custodianship or use; legitimacy requires demonstrable consent, accountability, and sustained engagement with affected communities.

By contrast, the NHA‐SA diverges from international standards by permitting state donation of non‐consensual unclaimed bodies, allowing institutions to legally acquire human deceased persons obtained without consent (Billings et al. 2024). The Ethics in Health Research Guidelines (South African National Health Department 2024; NDoH 2024), while setting high standards for biomedical research, provide no specific guidance on the use of anatomically‐derived human skeletonized individuals, creating further ambiguity. This diverges from international ethical norms and undermines the principle of informed consent, defined as voluntary, autonomous, and revocable (Nelson and Merz 2002; Fisher 2013; World Medical Association 2013; Laurijssen et al. 2022; Agarwal et al. 2024; Balta et al. 2025; Jones 2025).

Globally, opt‐in models (aligned with the Helsinki Declaration, World Health Organization 2011, and Singapore Statement 2010) require explicit donor consent, while opt‐out systems presume consent unless refusal is registered, although scholars warn that a lack of refusal does not constitute genuine consent (MacKay 2015; Kahn et al. 2017; Molina‐Pérez et al. 2022; Łuków 2023). In South Africa, the system of state donation resembles an opt‐out model, but one that lacks the cultural legitimacy required for validity. In this local context, formal burial and interment continue to be the dominant and deeply embedded practice, rooted in cultural, religious, and communal traditions that cut across diverse communities (Pike et al. 1991; Lee and Vaughan 2008; Dennie 2009; Gangata et al. 2010; Stig Sørensen and Rebay‐Salisbury 2013; Chidester 2014; Kramer et al. 2019; Masango 2005; De Gama et al. 2020; Hove et al. 2024; Jones 2024; Baliso et al. 2025). Mortuary culture has historically been shaped by belief systems, social organization, and historical circumstances, with burial established as the normative practice since at least the early 1900s and persisting as the preferred national mode of disposition today (Dennie 2003, 2009; Lee and Vaughan 2008; Lee 2011; Baloyi 2025). Beyond religious significance, burial serves as a means of maintaining ancestral ties, reinforcing social cohesion, and affirming communal belonging.

Political and historical forces also influenced these local practices. Funerary customs in Johannesburg, for instance, were used by urban South Africans as a way of reclaiming dignity in the face of colonial and apartheid oppression (Dennie 2009). Posel and Gupta (2009) emphasize that the treatment of the deceased body symbolizes the “social production of life,” embedding death practices in broader cultural and political meaning. Over time, mortuary practices adapted to processes of colonization, labor migration, the gold rush, apartheid, and circular migration, which reshaped the scale and expression of funerals (Lee and Vaughan 2008; Dennie 2009; Lee 2011; Baloyi 2025). During apartheid, the rise of burial societies provided an organized way to resist pauper burials and reclaim dignity in death, contributing to the development of elaborate funerary traditions in many South African urban communities (Dennie 2009). In this way, burial practices in South Africa are not only religious and cultural expressions but also forms of resilience, reclamation, identity, and resistance against structural inequalities.

Most NCAU deceased persons in South African repositories were acquired under apartheid, disproportionately involving bureaucratically defined “black‐African” and “colored” individuals who were more likely to die destitute and remain unclaimed after death (Gumede 2021; Mtapuri and Tinarwo 2021; Pellicer and Ranchhod 2023). Although legally sanctioned, the postmortem use of these skeletonized individuals without consent risks perpetuating structural violence and remains ethically concerning in post‐apartheid South Africa, where the effects of colonialism and systemic inequality persist (Halperin 2007; Alblas 2019; Forde et al. 2019; M'charek et al. 2020; Gumede 2021; Mtapuri and Tinarwo 2021; Kim and Friedlander 2023; Ntatamala et al. 2023; Pellicer and Ranchhod 2023). These structural vulnerabilities, such as health risks, poverty, oppression, systemic discrimination, and the risk of dying destitute, mean that these bodies should be treated as ethically sensitive to avoid further exploitation or harm (Halperin 2007; MacKay 2015; Forde et al. 2019; de la Cova 2020a, 2020b, 2022; M'charek et al. 2020; Zuckerman et al. 2021; Molina‐Pérez et al. 2022; Kim and Friedlander 2023; de la Cova et al. 2023; Agarwal et al. 2024). In health research, informed consent is foundational: it must be voluntary, informed, and revocable (Nelson and Merz 2002; Stoljar 2011; Fisher 2013; Tamariz et al. 2013; World Medical Association 2013; Sutrop and Lõuk 2020; Laurijssen et al. 2022; Agarwal et al. 2024; Meiring et al. 2024; Balta et al. 2025). Humanitarian frameworks therefore, suggest that individuals should retain control over how their bodies and tissues are used, both in life and after death, as an expression of their right to self‐determination (Jones 2019; Moon 2019), a legal and ethical principle that safeguards recognition, dignity, empowerment, and protection. Control over the body after death is about the social production of life (Posel and Gupta 2009). In contrast, NCAU deceased persons are non‐consensual and fall short of these ethical standards.

Securing consent from vulnerable communities is complex, and postmortem proxy consent raises further challenges. In some circumstances, representation of the deceased may be possible through family, community, or cultural associations, but such consent must be scrutinized to ensure alignment with the decedent's belief system and values (historical, social, class, race, or gendered contexts) (Pyburn 2011; Osuji 2018; Gibbon et al. 2024). In the case of NCAU bodies, often unclaimed, destitute, or acquired when records were incomplete, identifying appropriate representatives is often impossible. Proxy or relational autonomy consent requires demonstrable shared social, cultural, or religious ties (Stoljar 2011; Laurijssen et al. 2022). Decisions made solely by government regulators rarely meet these criteria, and unless a clear benefit to the decedent or their community can be shown, retrospective proxy consent may not be considered appropriate. Proxy consent in an ethics‐of‐care or relational autonomy framework is difficult to justify for NCAU individuals, particularly where community or familial associations cannot be established (Pyburn 2011; Osuji 2018; Smithsonian Human Remains Task Force 2024; Gibbon et al. 2024).

Stewards of skeletonized decedents through anatomical education programs in South Africa are faced with this dilemma of what is legal vs. ethical. In practice, through personal communication many South African institutions have moved away from accepting NCAU bodies. Where such deceased persons' bodies remain essential for anatomical education, tissue is not being retained or used for research, reflecting a shift toward IFAA‐aligned principles (Kramer et al. 2019; De Gama et al. 2020; Billings et al. 2024). Yet there are still large collections of NCAU skeletonized individuals that were acquired legally but now considered ethically problematic (Nystrom 2017; Fossheim 2019; Jones 2019). This ethical uncertainty surrounding the use of NCAU bodies in Africa, together with the reliance of anatomical education programs on such donations (Gangata et al. 2010; Chia and Oyeniran 2020; Matisonn and Muade 2023), creates challenges for custodians. Their continued use raises difficult questions: should they be retained, reburied, or cremated? Public trust in body donation programs depends on how institutions navigate this dilemma, balancing scientific value with dignity, cultural norms, and community expectations (Garment et al. 2007; Gangata et al. 2010; Champney 2011; Balta et al. 2025).

## 4 Decision Making, Retention and Disposition of NCAU Skeletonized Individuals Derived Through Anatomical Education Programs in South Africa

**FIGURE 1:**
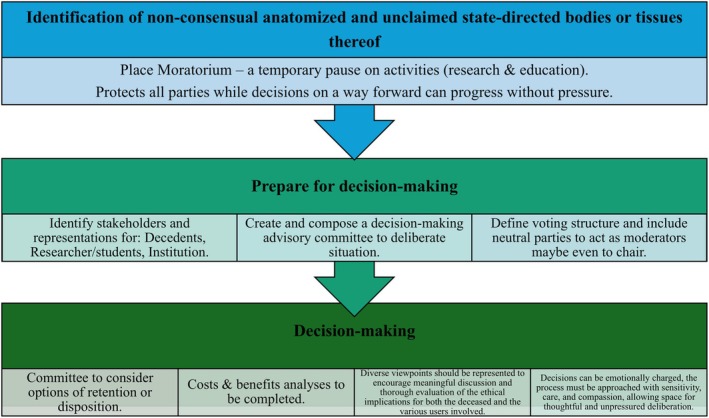
Proposed decision‐making pathway for the management of non‐consensual anatomized and unclaimed state‐directed bodies, comprising three sequential stages: Identification and moratorium, preparation for decision‐making, and final decision‐making by a multi‐stakeholder advisory committee.

When NCAU human skeletonized individuals are present, custodians find themselves navigating complex, multi‐faceted situations, grappling with the legal, ethical, and emotional dimensions of their stewardship. There are multiple stakeholders, and all positions should be considered during a robust, engaged decision‐making process. In this section, some considerations on ways to proceed are provided (Figure 1). When NCAU skeletonized individuals are identified, it is advised that they are placed under an immediate moratorium (Dunnavant et al. 2021; Black et al. 2022; Gibbon et al. 2024; de la Cova et al. 2024; Scholts 2024; Robbin Schug et al. 2025). This would be a temporary pause on their educational and research utility to the institution and its users, while decisions on a way forward can occur. It protects the deceased persons in the interim and removes pressure from the decision‐making process (Black et al. 2022; Gibbon et al. 2024; Scholts 2024; Robbin Schug et al. 2025). However, an indefinite moratorium risks leaving the decedents in prolonged limbo while imposing ongoing stewardship and storage demands on institutions without corresponding benefit, an unsatisfactory outcome for all. Placing an expiry date on the moratorium or setting an annual date for revisiting the status is advisable to ensure an active decision‐making and advisory process occurs.

Establishing a decision‐making committee would be an important and effective step to guide decisions, ensuring careful consideration of cultural, legal, and ethical aspects in managing such sensitive repositories (Champney 2011; Black et al. 2022; Gibbon et al. 2023). Table 1 provides considerations for establishing a committee and the value of setting out terms of reference, including establishing a voting structure before deliberation begins (Black et al. 2022; Cornwall et al. 2024; Gibbon et al. 2024).

**TABLE 1:** Considerations to establish a decision‐making advisory committee for non‐consensual anatomized and unclaimed state‐directed skeletonized individuals derived through anatomical education programs in South Africa.

Composition and size
Agree on a committee size to allow for composition, but not onerous to manage for meetings and voting.
Voting membership should be 5–9 people maximum, with balanced representation and an uneven number of voters.
Identify seats and whom members represent? How and why are they needed? What value do they add to the conversation? e.g., communities, ethicists, legal advisors, and scholars.
Balanced and objective membership to represent diverse perspectives.
Include members that do not benefit (neutral) from an outcome who can act as moderators.
Define who will chair and deputy/vice chair.
Provision for and identify external advisory expertise and input.

Membership/appointments
Should be related to representation or expertise.
Should have knowledge and expertise to relevant legislation, policies, procedures or guidelines to facilitate decision making.
Any conflict of interest must be declared.
Clarify rules for voting and non‐voting membership.
Define process of appointments, committee tenure and removal.
Proxy representation is expected whereby parties cannot attend or carry conflict of interest.

Meetings and rules of engagement
Define the scope aims and intentions of the meeting.
Agree on meeting frequency and mode (in‐person, hybrid, online).
Agenda, minutes and recording—agree on how discussions and consultations will be documented.
Identify accessible communication tools (email, WhatsApp, Teams, Skype, Zoom) etc. …
Define and deliberate membership conduct, behavior, professionalism and any confidentially/sensitivity if required.
Define voting quorum.
Awareness that back‐and‐forth communication can require time for external consultation, factoring in reasonable time.
How will decisions be made? When can the appointed voting person make decisions and when will broader consultation be required?
Establish modes for conflict resolution.
*Aspects of the table have been influenced by* Black et al. (2022), Gibbon et al. (2024), Champney (2011), Cornwall et al. (2024)

Most repositories of human skeletonized individuals in South Africa (heritage or anatomical) use decision‐making committees in addition to some requiring Human Research Ethics Committee permission and approvals (Black et al. 2022; Baliso et al. 2025). To discuss ethical concerns for decedents derived through anatomical education programs, a decision‐making committee should be established. It would be advantageous to have an ethicist, a geneticist/molecular specialist, a biological anthropologist or two, an anatomist, and other experts who work with the deceased, such as a forensic pathologist, and archaeologists. Someone from the social sciences, such as a social or cultural anthropologist, historian, or someone from African studies, would be useful. Additionally, as recommended by the Smithsonian Human Remains Task Force (2024), a member representing relational proxies (broadly defined communities) in decision‐making. It is essential that the committee be chaired by a neutral individual who has no stake in the outcome, ensuring an objective and balanced process. Diverse viewpoints should be included to support meaningful discussion and a thorough assessment of the ethical implications for both the deceased and the users involved.

Because these decisions can be emotionally charged, the process should be approached with sensitivity and allow space for careful, unpressured deliberation. Such a committee would help address the marginalization, and the interdisciplinary perspectives provide critiques not typically offered by scientists while generating advice that can inform policy.

Outlined below are considerations for the retention or disposition of NCAU skeletonized individuals, summarized in Tables 2 and 3, without advocating for a specific course of action. Each case is shaped by its unique context and institutional culture, making a one‐size‐fits‐all solution impractical. Engagement from students, communities, and a broad range of institutional stakeholders may be necessary. Ultimately, what matters most is fostering open, evidence‐based dialogue and ensuring that decisions are grounded in facts, guided by local, national, international, and institutional frameworks.

**TABLE 2:** Considerations of options for decision‐making advisory committees regarding the retention and disposal of non‐consensual anatomized and unclaimed (NCAU) state‐directed skeletonized individuals derived through anatomical education programs in South Africa.

Retention options	
Temporary/transitional retention	
Retention under moratorium	Cease active use (moratorium) until consensus is built around ethical frameworks (decision‐making committee see Table 1).
Temporary retention for future of ethical replacement	Retention is a short‐term process while unethical legacy repositories are replaced by fully consented donors. Thus, future educational and research needs will be ethical. Although these donor‐only collections may suffer demographic bias, disproportionately representing populations more willing or able to donate (Kramer et al. 2019).
Permanent retention	
Retention with restricted access and use guidelines	Institutions with sufficient capacity for long‐term, respectful care and secure storage may justify retention where ethical concerns are mitigated. This includes restricted access for education, controlled by ethics committees, with clear documentation, oversight, and limits set on handling and display. For example, retention of rare or pathologically unique embodiments may be irreplaceable for education and research tools.
Grandfather clause for legacy collections	Retain NCAU human skeletonized individuals obtained legally but now under ethical scrutiny, as they are irreplaceable to education and research, this requires transparent documentation institutional review (decision‐making committee), and community consultation. Acknowledges the decedent's contributions to education and research without physical reinterment, e.g., inclusive of plaques, memorial displays, or digital remembrance.
Retention for memorialization and humanitarian purposes	Repositories of NCAU human skeletonized individuals become memorial spaces that honor the lives and contributions of these individuals to health sciences education and research, especially when consent was absent.
Ethics‐educational resource	Retained NCAU human skeletonized individuals could serve anatomical education, but as real‐world educational tools for bioethics, law, and history of science curricula.
**Disposal options**	
Disposal should not reinforce or create new human rights violations (Moon 2019; Tyagi and Rathee 2024; Yadav 2007). Widespread disposal of NCAU human skeletonized individuals risks erasing irreplaceable data, particularly from underrepresented population groups (Kramer et al. 2019; Baliso et al. 2025).	
Cremation	Legally recognized under South Africa's National Health Act, anatomically‐derived human body donors. Efficient and standardized. May conflict with cultural/religious norms. Respect for posthumous religious and cultural beliefs must be considered, even if specific data are unknown. This is particularly critical in South Africa, where cremation may conflict with traditions that view the body as transitioning into the role of an ancestor (Glass and Samuel 2011; Hove et al. 2024).
Restitution to families or communities	Where identity or origin is known or can be reasonably inferred, pursue respectful restitution in collaboration with relational autonomy representatives (e.g., community) (Gibbon et al. 2024).
Community‐informed reburial or cremation	Guided by traditional, religious, or cultural practices relevant to the individuals or their presumed communities. Where cultural norms are unknown, regional normative burial practices should be used as a proxy standard (De Gama et al. 2020).
Symbolic burial or memorialization	Group memorial services or symbolic interments may provide respectful closure.
Communal/mass burial	Used in public mortuary systems for unidentified or destitute individuals. May be seen as undignified (due to shortage of burial ground) or lacking ritual.
Individual burial with ceremony	Considered best practice in South African heritage sector (Black et al. 2022). Includes coffin, grave, and ceremony. Most culturally respectful, but costly (~ZAR 1 million/€50,000).
Digital preservation prior to disposal	Digital preservation (3D scanning and 3D printing) may preserve anatomical data for future educational use before disposal. However, this too requires ethical oversight, especially for remains obtained without consent (Cornwall 2016; L’Abbé et al. 2024).

**TABLE 3:** Considerations for risks and benefits analyses for decision‐making advisory committees regarding the retention and disposal of non‐consensual anatomized and unclaimed (NCAU) state‐directed skeletonized individuals derived through anatomical education programs in South Africa.

Decedent
Human rights to dignity, respect and self‐determination in life and after death.
Is a case for normative opt‐in consent arguable?
Are living people to act as relational autonomy proxy or next‐of‐kin consent traceable or identifiable? Thus, allowing for retrospective proxy consent.
Can a case be made that state consent was provided as the person was structurally vulnerable?
Does the health sciences education or research benefit the decedent or their relational descendants?
Is there risk to the decedent or their relational descendants for continued use in health sciences education and research?
A pillar of research is consent; can they be retained for education but not for research?
Socially and culturally normative concepts of ethical body retention or disposal.

Researcher/student
Does the research need outweigh the decedent's rights to consent?
Can data being sought be obtained elsewhere, either in the nation or internationally?
Can the data be supplemented statistically?
Has the research been designed in an ethically considerate manner?
For degree purposes effect of using a known unethical sample on the examination process.
Effect of long‐term professional trajectory and employment after using an ethically concerning or otentially unethical sample for research.
Is the use of known NCAU human skeletonized individuals, which can be classified as a ethically concerning standard practice for research in the discipline, currently nationally and internationally?
Has the status of the sample changed mid‐study or degree? Thus, impacting an already approved study.
Are the research questions and study scope clearly defined?

Institution
Is there potential for societal backlash or social outcry for use?
Cost implications for retention and disposal?
If a student or researcher was permitted access and then subsequently fails examination or fails to become employed because of the decision is there a chance of lawsuit?
Reputational harm to the institution and its educational and research practices?
Is the use of NCAU human skeletonized individuals aligned with institutional ethical practices, guidelines, policies and ethos?
What are the risks, benefits and significance of this research for all parties?
How does the institution tangibly benefit from this research?

### 4.1 Retention of NCAU Skeletonized Individuals

It is well documented nationally and internationally that repositories of human skeletonized individuals derived through anatomical education programs contribute to education, science and research. They are often regionally specific (Petaros et al. 2021; Baliso et al. 2025), with well‐documented historical and biological records serving as invaluable scarce resources (Campanacho et al. 2021). Despite the wealth of knowledge housed within these repositories, their use is accompanied by significant limitations and ethical challenges. Issues such as limited accessibility, threats to long‐term preservation, and a general lack of awareness about their existence have contributed to some institutional collections being underutilized (Cornwall et al. 2012; Adana et al. 2019; Bookholane et al. 2020; De Gama et al. 2020; Manjatika et al. 2024; Meiring et al. 2024). At the heart of our work as curators, is a deep commitment to serving humanity, both the living and those who came before us. As Brauer (1992) eloquently stated, the goal of research conducted in repositories of human skeletonized individuals is to confront the challenges facing humanity today while preserving our collective heritage. The human skeletons originating from anatomical education programs are entrusted to us to carry more than just biological data, they represent real lives, histories, and identities. With known demographics such as age‐at‐death, sex assigned at birth, height, cause‐of‐death, and in some cases, reported race or ethnicity, these individuals offer insights into the human condition (Brauer 1992; Albanese 2003; Hunt and Albanese 2005; Cornwall and Stringer 2009; Champney 2011; Alblas 2019; Belcastro et al. 2022). This known information allows for the creation of population‐specific standards that are essential for understanding human biology, health, disease, lifestyle, and how populations have changed over time (Buikstra and Ubelaker 1994; Hunt and Albanese 2005; Alblas 2019; Hagelthorn et al. 2019; Belcastro et al. 2022; Kaledzera et al. 2023). Such standards are not just academic exercises; they are practical tools that help support and protect the living. In forensic anthropology, these data help law enforcement identify unknown individuals, bringing closure to families and justice to communities (Campanacho et al. 2021; Gibbon and Morris 2021). As national methods became refined, national repositories grew, so too did the understanding of the complex biological, cultural, and historical stories embodied in deceased individuals. These repositories are invaluable for education and training, giving students the opportunity to engage with real human tissue in a way that fosters empathy, curiosity, and respect (Winkelmann 2016; Gibbon and Morris 2021).

Kahn et al. (2017) discuss the incompatibility of using decedent bodies that were unclaimed state‐directed in ethical anatomical education or research. Global trends in human body donation show fluctuation, with some countries, such as Africa and Asia (Singh Chauhan 2026; Oloya et al. 2026), reporting severe shortages of willing donors, while other countries, such as the USA, have regional shortages in some areas and maintain stable donor programs in others (Kalter 2019). The South African context similarly reflects variation, with some institutes noting low uptake with consenting donation (Masango 2005; Lee and Vaughan 2008; De Gama et al. 2020; Kramer et al. 2019), and other institutes indicating increased engagement (Billings et al. 2024; Matshipi and De Gama 2024). Jones (2019) provides an utilitarian ethics based an argument for retention in service to the community and humanity, and a compromise may be required. A consideration is whether the resource can be replaced with donations from contemporary consented individuals' overtime, acknowledging this may be gradual as it depends on donor permissions (Meiring et al. 2024). Within this context, the argument that the destruction or disposal of legacy collections constitutes a loss of valuable scientific resources must be approached with caution. If so, the collection could align with both legal and ethical standards. Another consideration involves repositories of human skeletonized individuals with unique or rare pathological or traumatic features, where their retention for educational purposes may need to be carefully weighed against ethical concerns. A possible approach might involve implementing a grandfather clause with specific guidelines on use, handling, and acknowledgement of ethical considerations in both research and educational contexts.

### 4.2 Disposition of NCAU Skeletonized Individuals

Some view the disposition of NCAU bodies as a respectful and harm‐minimizing solution. However, Jones (2019) argues that such decisions are not ethically neutral, as disposal removes these deceased persons from service to both the institution and humanity. Others raise concerns about violations of self‐determination when retention or disposal occurs without clear consent (Yadav 2007; Moon 2019; Tyagi and Rathee 2024), highlighting the diversity of perspectives.

If retention is ruled out, what options of disposition are ethically and culturally appropriate? The NHA‐SA prescribes cremation for anatomically deceased persons and remains thereof, a practice also followed in India (Charmode et al. 2020). Cremation is currently the standard practice for anatomical donation programs in South Africa, this was brought to the country by migrants from India of the Hindu faith who fought for this religious practice (Dennie 2003). It was later adopted by European South Africans with adaptations to the local religious practices and standards and gained traction (Dennie 2003; Chidester 2014). The cost saving and reduction in land requirement resulted in it being adopted by the South African government (during the colonial and apartheid periods) as the standard practice for body disposition by the state for anatomical programs, and is retained in the current legislation (Dennie 2003). Conversely, unidentified or destitute deceased persons in the mortuary system that are not donated for educational purposes are often buried in communal graves containing three to five persons at the expense of the government (Naidoo 2007; Wild 2017). Some argue that the institutional stance to refuse transfer or acceptance of NCAU bodies places a financial burden on the government and consumes valuable and scarce land resources for burial. Therefore, NCAU bodies can be argued as nationally beneficial to society and health sciences education and may reduce strain on public resources. For many cultural and religious groups, such practices are considered undignified and contrary to deeply held beliefs (Masango 2005; Lee and Vaughan 2008; Dennie 2009; Gangata et al. 2010; Lee 2011; Chidester 2014; De Gama et al. 2020; Hove et al. 2024; Jones 2024; Baliso et al. 2025; Baloyi 2025).

In isiZulu culture, burial on family land is essential for reintegration into the lineage of *amadlozi* (ancestors), and communal graves are rejected as they disrupt ancestral ties and prevent crucial rituals such as *ukubuyisa* (bringing the spirit home) and cleansing ceremonies (Pelewe et al. 2025). Similarly, among isiXhosa communities, burial is a vital rite of passage into the ancestral realm, and mass or anonymous graves are regarded as spiritually harmful (Mbiti 2015; Mulambuzi 1997). Within Islam, cremation is strictly forbidden, and communal burial without individual recognition undermines the dignity required for the decedent, who must be buried as soon as possible facing Mecca (ICRC 2020). Across these groups, disturbance, displacement, or undignified disposal of the body undermines social and spiritual integrity.

While cremation is standard practice for consenting donors, cultural and religious beliefs in South Africa often reject it, favoring the body's return to the earth through burial. Since the beliefs and customs of NCAU decedents are frequently unknown (De Gama et al. 2020), normative regional practices that treat death as a transition and emphasize burial rites for ancestral acceptance may be more appropriate (Masango 2005; Lee and Vaughan 2008; Glass and Samuel 2011; Hove et al. 2024). These cultural perspectives remain a significant factor contributing to South Africa's low body donation rates (Lee and Vaughan 2008; Masango 2005; De Gama et al. 2020; Billings et al. 2024; Matshipi and De Gama 2024).

As with retention, a risk–benefit analysis should guide disposal decisions, accounting for ethical, sociocultural, and financial factors. Notably, retention and proper stewardship are not cost‐neutral; thus, costs will be incurred regardless of retention or disposal, and they must be anticipated from the outset.

### 4.3 Decision‐Making

Decision‐makers must weigh sociocultural expectations against human anatomical discipline norms. Best ethical practice requires that institutions move beyond risk assessments and adopt forms of community consultation and participatory decision‐making. For example, the Sutherland Nine restitution process (Gibbon et al. 2023) was achieved through community collaboration. Recent international guidance offers models: the Smithsonian Human Remains Task Force (2024) recommends structured listening sessions with affected communities, while the American Anthropological Association's Commission (Agarwal et al. 2024) advocates for transparent provenance reporting and opportunities for communities to shape outcomes. Applied to South Africa, this could involve collaboration with cultural, religious, and community leaders in areas historically associated with NCAU skeletonized individuals. Even when individual descendants cannot be identified, broader cultural or faith communities can act as legitimate stakeholders in decisions regarding retention, research, or respectful reburial in a form of relational autonomy consent (Lee and Vaughan 2008; Gibbon et al. 2024).

In heritage restitution practice, individual burials with coffins and ceremonies are standard and can cost over a million Rand (~€50,000) per grave (Black et al. 2022), necessitating careful budgeting. Community consultation is also important (Gibbon et al. 2023; Agarwal et al. 2024), though this may not always be feasible for unidentified decedents. In cases where cultural identity can be inferred, for example, Islamic identifiers, consulting local religious leaders may enable context‐sensitive decisions. Additionally, limiting body donations to specific cultural or religious groups may skew the representative nature of repositories of human skeletonized individuals, reducing their representativeness for research relevance and societal impact. Institutions moving to donor‐only models risk underrepresenting South African majority populations, resulting in loss of population‐specific data (Kramer et al. 2019; Baliso et al. 2025), which could hinder both education and socially impactful research.

Given these complexities, a thorough risk–benefit analysis is essential. It must weigh research and educational importance against the rights and dignity of the deceased. As those who benefit from these skeletonized individuals, it is important to ask: Is their use truly ethical, regardless of how invaluable they are to our work? While the answer is uncertain, our duty lies not only with research and education, but with the communities, past, present, and future, that we aim to serve.

## 5 Institutional Support for Custodians to Execute Stewardship and Curation

This section is an auto‐ethnographic account as curators within South Africa, as well as members of the South African curator collective (composed of curators of human skeletonized persons providing a national representation of South African museums and institutions of higher education) and the Anatomical Society of Southern Africa, the largest national professional association of biological anthropologists and anatomists. Curators are frequently involved in institutional decision‐making processes, placing them in positions of both authority and responsibility (vulnerability). Through our professional experience, concerns have emerged regarding the potential misuse of curatorial authority for career advancement and the promotion of collaborative networks, nationally and internationally. These concerns may include preferential access to collections for selected researchers or the inappropriate attribution of authorship through access provision to deceased individuals. In our view, curatorship is understood as a role requiring a balanced approach to research and education, grounded in stewardship and custodianship of the deceased individuals in their care.

From a curatorial perspective, the stewardship of repositories of human skeletal remains, particularly those derived through state and anatomical education programs, has become increasingly complex. There is widespread recognition that many of these repositories originated through ethically problematic practices associated with structural marginalization and historical injustice (Jones 1994; Sealy 2003; Alves‐Cardoso 2019; de la Cova 2020a, 2020b, 2022; Squires et al. 2020; Campanacho et al. 2021; IFAA 2022; Black et al. 2022; de la Cova et al. 2023, 2024; Kim and Friedlander 2023; Stantis et al. 2023; Agarwal et al. 2024; Agarwal 2024; Rassool and Gibbon 2024; Smithsonian Human Remains Task Force 2024). Although the decisions that shaped these repositories were made historically, their contemporary management now occurs within the context of increased public scrutiny and ethical accountability, requiring careful consideration of research value, educational significance, and the respectful treatment of the deceased. In practice, these responsibilities can create professional uncertainty. Continued use of such repositories may attract criticism regarding ongoing engagement with ethically contentious collections and complacency (Walters and Jansen 2022), whereas advocating for their respectful disposition may have professional or institutional implications, such as jeopardizing careers, reputations, and institutional relationships. Such critiques are often interpreted within broader debates concerning racism, colonial legacies, and institutional accountability. In some observed instances, curators have been removed from their curatorial positions for defending ethical stances in collection management. Without institutional protection or legal clarity, many curators live with the constant fear of being scapegoated if decisions provoke backlash on the institution. As Black et al. (2022) note, curators are navigating shifting sands, forced to make significant ethical decisions with rapidly evolving professional and social contexts.

These challenges are further compounded by the limited availability of formal training, institutional recognition, and resources for curatorial work. In South Africa, curators of human skeletonized individuals within universities frequently lack formal training in curatorship. In many cases, curation and stewardship responsibilities are expected of lecturers and professors on top of their already demanding academic duties, contributing to further capacity constraints. This may include insufficient time within standard working hours to complete curatorial responsibilities effectively. Many curators acknowledge that they operate within under‐resourced and capacity‐limited environments. Conditions that may also limit opportunities to remain current with developments in ethics and curatorial management, or to implement best practices where financial resources are insufficient. The considerable funding required for the processing, cataloguing, storage, and preservation of skeletonised persons and their associated documentation remains a substantial challenge for maintaining large repositories (Albanese 2003).

The absence of sustained institutional support, including financial, structural, and legal support, can make curatorial responsibilities particularly challenging and, at times isolating. In contexts where institutional leadership and accountability structures are still developing, curators may carry substantial ethical and reputational responsibilities, despite the collective institutional implications of these decisions. Policies require that matters involving the deceased be managed by trained curators with archaeological or anthropological expertise (Baliso et al. 2025), yet implementation of these mandates depends on meaningful institutional support. Proactive institutional leadership that provides guidance, trust, and protection is needed, ensuring that decisions about ethically complex collections are made collaboratively, transparently, and with foresight, rather than placing undue responsibility on individual curators. Within South Africa, leadership in this domain is still developing across institutions, with opportunities to strengthen both depth and consistency.

## 6 Conclusions

Curators hold the important responsibility of navigating the complex ethical, cultural, and scientific landscapes surrounding legacy collections of human skeletonized individuals. Ultimately, decolonizing forensic anthropology and clinical anatomy requires more than diversifying content; it demands a paradigm shift in how curatorship is conceptualized and enacted. This involves reframing repositories of NCAU human skeletonized individuals not as neutral objects of science, but as legacies of structural marginalization and vulnerability whereby ethical stewardship must contend with historical violence and its ongoing repercussions. By critically engaging with these controversies of curatorship, South African institutions can contribute to a global reimagining of ethical practice, one that is attentive to both historical accountability and future responsibility. By addressing their difficult histories and reimagining their potential, we can ensure these repositories are used ethically, responsibly, and respectfully in contemporary educational and research contexts. The challenge lies in deriving valuable insight without perpetuating past harms.

This commitment not only preserves the dignity of the individuals represented but also deepens our understanding of humanity's shared history. To achieve this, institutions must lead by developing clear legislation, offering robust support, and fostering a culture grounded in ethical responsibility. A structured, multidisciplinary approach is advocated for ensure transparent and ethically responsible stewardship in navigating challenges related to the curation of these NCAU repositories. The complexities surrounding their retention, use, or disposition are discussed, and guidelines are proposed, including a moratorium to allow time for inclusive dialogue, committee formation, and consideration of strategies for ethically informed and inclusive decision‐making. While the focus here is on NCAU skeletonized individuals the paper is applicable to all NCAU decedent bodies and parts thereof, which may reside in institutional spaces. Continued dialogue, collaboration, and visionary leadership are essential to creating an environment where curators can make informed, ethically sound decisions without fear of professional or legal repercussion. Furthermore, institutions should prioritize professional development, training, and certification programs for curators to equip them with the tools to navigate these sensitive issues with integrity and confidence. In South Africa, in the absence of an ethical position under the legislation, contending with these controversial collections and their direction in the future can only be overcome collectively with consideration for multifaceted and complex perspectives and will involve robust and even difficult conversations. Engaging in these conversations takes the direction toward transformation.

## Author Contributions

**A. Alblas:** conceptualization, writing – original draft, investigation, writing – review and editing. **V. E. Gibbon:** conceptualization, writing – original draft, investigation, writing – review and editing.

## Funding

V.E.G. received funding support from the South African National Research Foundation.

## Ethics Statement

The authors have nothing to report.

## Conflicts of Interest

The authors declare no conflicts of interest.

## Supporting information


**Figure S1:** ajpa70300‐sup‐0001‐FigureS1.png.

## Data Availability

Data sharing is not applicable to this article as no datasets were generated or analyzed during the current study.
