# Supplementary figures and images for: Custodians of Controversy: Navigating Stewardship Challenges With Non‐Consensual Anatomized Human Skeletonized Individuals in South Africa
Source: Am J Biol Anthropol. 2026 Jul 2;190(3):e70300. doi: 10.1002/ajpa.70300 (PMC13329213; doi:10.1002/ajpa.70300)

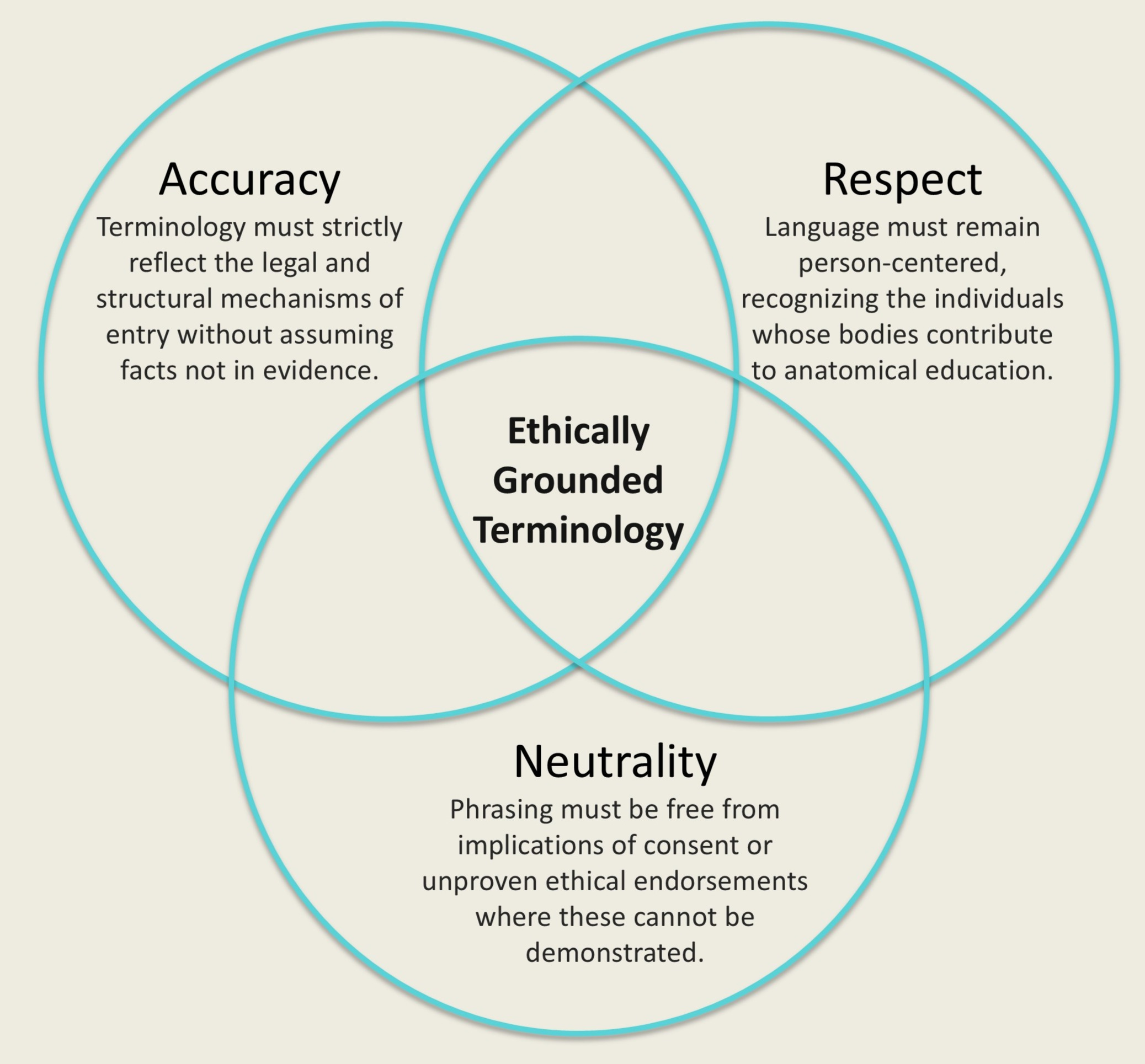

Supplement: Supplementary file 1 — Figure S1: ajpa70300‐sup‐0001‐FigureS1.png. [file AJPA-190-e70300-s001.png]
